# An algorithm for automated modulation transfer function measurement using an edge of a PMMA phantom: Impact of field of view on spatial resolution of CT images

**DOI:** 10.1002/acm2.12476

**Published:** 2018-10-19

**Authors:** Choirul Anam, Toshioh Fujibuchi, Wahyu Setia Budi, Freddy Haryanto, Geoff Dougherty

**Affiliations:** ^1^ Department of Physics Faculty of Mathematics and Natural Sciences Diponegoro University Semarang Central Java Indonesia; ^2^ Department of Health Sciences Faculty of Medical Sciences Kyushu University Fukuoka Fukuoka Prefecture Japan; ^3^ Department of Physics Faculty of Mathematics and Natural Sciences Bandung Institute of Technology Bandung West Java Indonesia; ^4^ Applied Physics and Medical Imaging California State University Channel Islands Camarillo CA USA

**Keywords:** automated MTF calculation, edge spread function, field of view, modulation transfer function, PMMA phantom, spatial resolution

## Abstract

**Purpose:**

The purpose of this study was to introduce a new algorithm for automated measurement of the modulation transfer function (MTF) using an edge of a readily available phantom and to evaluate the effect of reconstruction filter and field of view (FOV) on the spatial resolution in the CT images.

**Methods:**

Our automated MTF measurement consisted of several steps. The center of the image was established and an appropriate region of interest (ROI) designated. The edge spread function (ESF) was determined, and a suitably interpolated ESF curve was differentiated to obtain the line spread function (LSF). The LSF was Fourier transformed to obtain the MTF. All these steps were accomplished automatically without user intervention. The results of the automated MTF from the edge phantom were validated by comparing them with a point image, and the results of the automated calculation were validated by the standard fitting method. The automated MTF calculation was then applied to the images of two polymethyl methacrylate (PMMA) phantoms and a wire phantom which had been scanned by a Toshiba Alexion 4‐slice CT scanner and reconstructed with various filter types and FOVs.

**Results:**

The difference in the 50% MTF values obtained from the edge and point phantoms were within ±4%. The values from the automated and fitted methods agreed to within ±2%, indicating that the automated MTF calculation was accurate. The automated MTF calculation was able to differentiate MTF curves for various filters. The spatial resolution values were 0.37 ± 0.00, 0.71 ± 0.01, and 0.78 ± 0.01 cycles/mm for FC13, FC30 and FC52 filters, respectively. The spatial resolution of the images decrease linearly (*R* ^2^ > 0.98) with increasing FOVs.

**Conclusion:**

An automated MTF method was successfully developed using an edge phantom, the PMMA phantom. The method is easy to implement in a clinical environment and is not influenced by user experience.

## 1 INTRODUCTION

A CT scanner is an effective and efficient tool to obtain quality images of patients for diagnosing diseases and abnormalities.^1^, ^2^ Image quality is determined by several parameters, including spatial resolution,^3^ noise,^4^, ^5^ and low contrast detectability.^6^ Some of these parameters should be monitored through periodic quality control.^7^

Spatial resolution is a measure of the ability to differentiate adjacent objects in an image.^8^ It is important to evaluate spatial resolution carefully, since objects in a CT image are corrupted by the point spread function (PSF) of the CT imaging system.^9^ The PSF is affected by factors such as the finite size of the x‐ray source^10^ and the limitations of the image reconstruction algorithm, e.g. filtered back‐projection (FBP) method or iterative reconstructive (IR) method.^11^ The accuracy of measuring small densities or thin structures within the body depends on the spatial resolution.^9^ In practice, the spatial resolution is determined based on the ability to discriminate a line pair object in a phantom.^12^, ^13^ However, this conventional technique is very subjective. An objective way to characterize the spatial resolution is by using the modulation transfer function (MTF) curve.^14^, ^15^, ^16^

There are several methods for determining MTF: using point spread function (PSF),^17^ line spread function (LSF),^18^ edge spread function (ESF)^19^ and directly from image bar patterns (BP).^20^, ^21^ The MTF calculation can be carried out analytically using ESF, PSF, or LSF, i.e. by converting the ESF to the LSF by differentiation,^19^ or by converting the PSF to LSF by an averaging process^17^ and then taking the Fourier Transform of the LSF.^17^ The calculation of the MTF can also be performed by using a fitting method either with the LSF curve^22^ or with the ESF curve,^23^ or calculated directly from phantom bar patterns.^20^ The calculation of MTF using these methods is tedious, time consuming and highly dependent on the expertise of the medical personnel involved. The calculation speed and objectivity of MTF calculations can be increased using automation with appropriate software.^24^

Up until now, MTF calculations (manually or automatically), based on PSF, LSF, ESF, or BP, require specific phantoms such as the AAPM CT performance phantom,^25^ Catphan phantom,^21^, ^26^ ACR CT accreditation phantom,^19^ MHT‐type phantom^27^ or a specially designed edge phantom.^18^ However, these phantom types may not be owned by a CT center, especially in developing countries. The type of phantom most probably owned by the CT center is a phantom to measure the CT output dose (CTDI_vol_), the polymethyl methacrylate (PMMA) phantom.^28^ For this reason, we developed automated MTF measurement using the PMMA phantom. We then implemented our automated MTF calculation method to evaluate the effect of reconstruction filter and FOV on spatial resolution.

## 2 MATERIALS AND METHODS

### 2.A Automated MTF measurement

**Figure 1:**
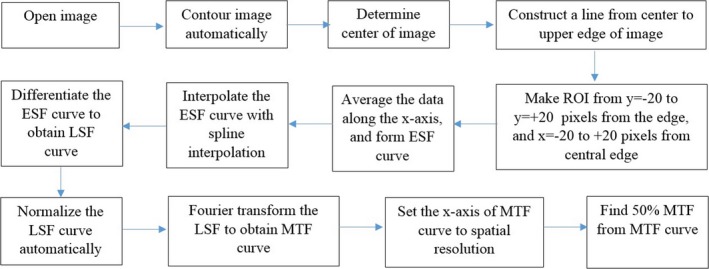
The steps of the automated MTF calculation.

The steps of the automated MTF calculation are shown in Fig. 1. These steps were implemented in MatLab (Mathworks Inc., Natick, MA Natick, MA) and the user simply uses a single button to complete them. We used standard netbooks for computation (Intel Celeron CPU 1005M, 1.90 GHz, installed RAM 2.0 GB, and 32‐bit operating system). The CT image was opened in its original DICOM format. The phantoms were contoured automatically,^29^, ^30^ and the center of the image was determined using the centroid equation.

(1) $$\left(x_{c},y_{c}\right)=\frac{1}{N}\sum_{i=1}^{n}\sum_{j=1}^{n}\left(x_{i},y_{j}\right)$$

**Figure 2:**
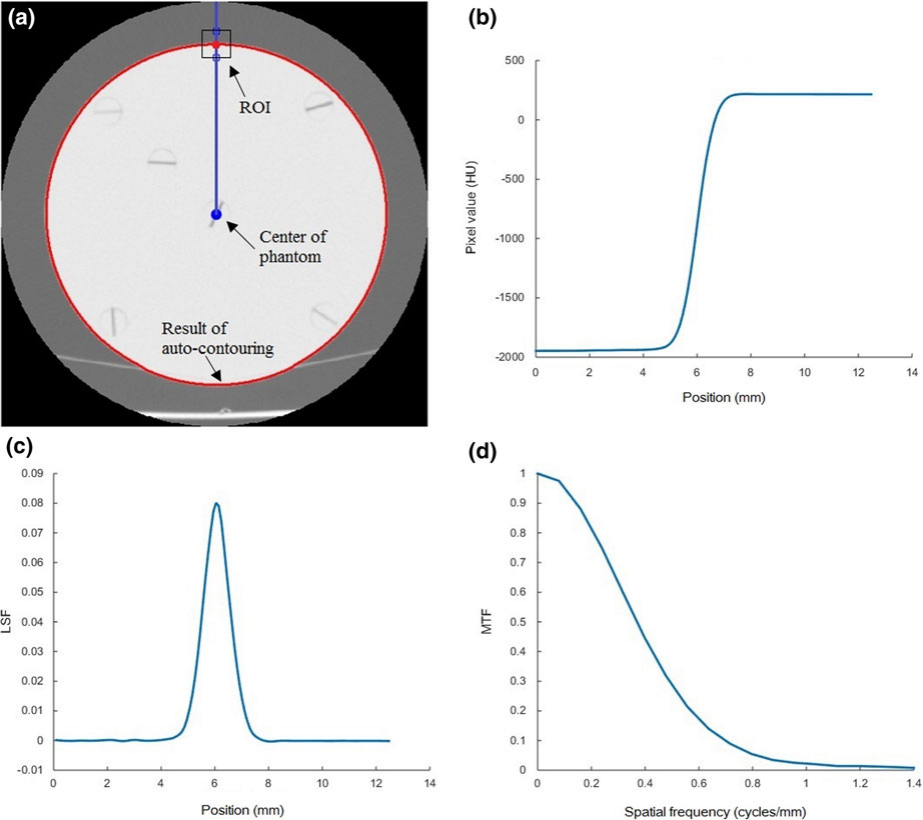
(a) A contoured image and the ROI at the upper edge used to calculate the MTF. (b) The interpolated ESF curve. (c) The LSF curve after the zeroing and normalization processes. (d) The resulting MTF curve used to characterize the spatial resolution of the image.

A line was constructed from the center of the phantom to the top of image, so that it passes through the upper edge of the image. This position is considered the center of the region of interest (ROI), with coordinates (*x*_c_, *y*_c_). A ROI was created with *x* running from *x*_c_ − 20 pixels to *x*_c_ + 20 pixels, and y from *y*_c_ − 20 pixels to *y*_c_ + 20 pixels [Fig. 2(a)].

The averages of the *x* values in the ROI were taken to give the edge spread function (ESF) where the *x*‐axis is the position of each pixel upward and the *y*‐axis is the value of each pixel. This ESF curve under‐sampled the data because the distance between pixels was relatively large.^31^ For example, for the head PMMA phantom with a 20 cm FOV the pixel size is 0.39 mm. We interpolated the data using spline interpolation to obtain four additional data points between each pixel, in order to secure a higher spatial resolution in the resulting MTF. The interpolated ESF is shown in Fig. 2(b).

The ESF curve was differentiated to obtain the LSF curve, which was then zeroed and normalized. The zeroing process forces the LSF tails to zero values, by subtracting the average of the five pixels in the left‐most part of the curve from all the values of the curve. Normalization of the LSF was required in order to fix the MTF value at 1 for a spatial frequency of 0, after the LSF is converted to MTF. Normalization was done by dividing the LSF by the sum of its original values. The resulting LSF curve, after zeroing and normalization, is shown in Fig. 2(c).

The LSF curve was Fourier transformed to obtain the MTF curve using:

(2) $$X\left(k\right)=\sum_{j=0}^{N-1}x\left(j\right)e^{(-i2\pi kj)/N}$$

where *k = *0, *…*,* N − *1 and *N* is the vector length of the LSF curve. The spatial frequency of the MTF curve in the *x*‐axis is given by:

(3) $$\omega_{s}=\frac{1}{N_{I}\times I_{s}}$$

where *N*_I_ is the number of pixels (in the *x* or *y* direction, respectively) and *I*_s_ is the sampling interval obtained from:

(4) $$I_{s}=\frac{\mathrm{FOV}}{512}$$

A typical MTF curve is shown in Fig. 2(d), from which the spatial resolution (viz. the spatial frequency at an MTF value of 50%) can be determined.

### 2.B Phantom and data acquisition

Two PMMA phantoms were used in this study, one with a diameter of 16 cm and the other with a diameter 32 cm.^29^ The PMMA phantoms are usually used to represent the index of the absorbed dose for adult head/abdomen.

The PMMA phantoms were placed on the patient table at different positions. Usually, the holes are positioned at 3, 6, 9, and 12 o'clock, but in this study the holes were positioned at 45° from their original positions. This is because the edge to measure the MTF uses the upper edge of the phantoms, so that such a phantom placement guarantees homogeneity within the phantom close to the upper edge. Conversely, if the phantom placement is not rotated 45°, there is an inhomogeneity within the phantom near the upper edge at the small hole used to place the ionization chamber pencil, and so it could not be used to measure MTF. Notwithstanding, the phantom position can be used to calculate the MTF using a special procedure called tail replacement on the resulting ESF curve as proposed by Sanders et al.^3^

**Figure 3:**
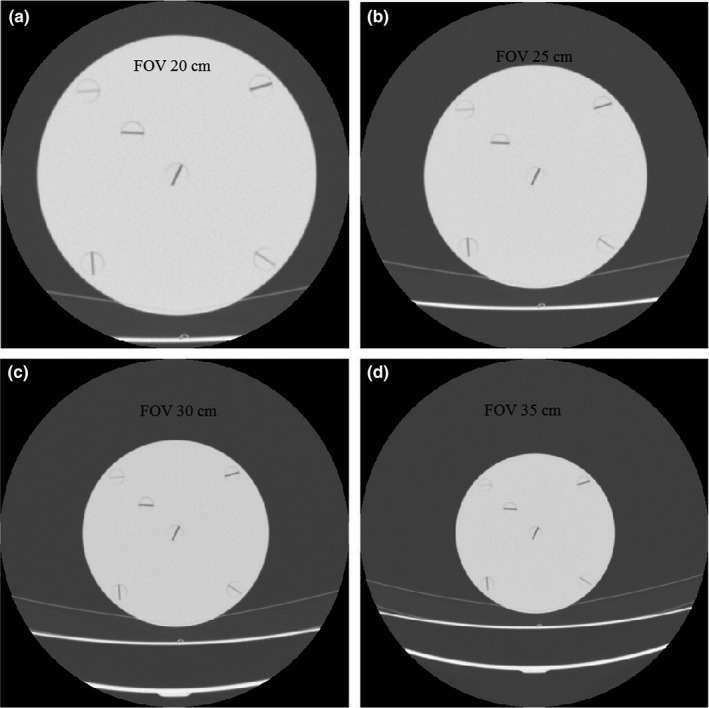
Examples of head PMMA phantom images for various fields of view. (a) 20 cm, (b) 25 cm, (c) 30 cm, and (d) 35 cm.

In this study, the PMMA phantoms were scanned using a Toshiba Alexion 4‐slice CT scanner. The scan parameters are indicated in Table 1. The phantoms images were reconstructed using three filter types, namely FC13, FC30, and FC52. FC13 is a filter used for soft tissue, FC30 for bone, and F52 for lung. For the head PMMA phantom, the field of view (FOV) was varied (Fig. 3), while for the body PMMA phantom we used a FOV of 35 cm.

**Table 1:** Scan parameters

Scan parameter	Setting
Tube voltage	120 kVp
Rotation time	1 s
Slice thickness	2 mm
Nominal beam width	4 × 2 mm
Focal spot	1.1 × 1.1 mm
Filter type	Large

### 2.C Validation of using the edge phantom

**Figure 4:**
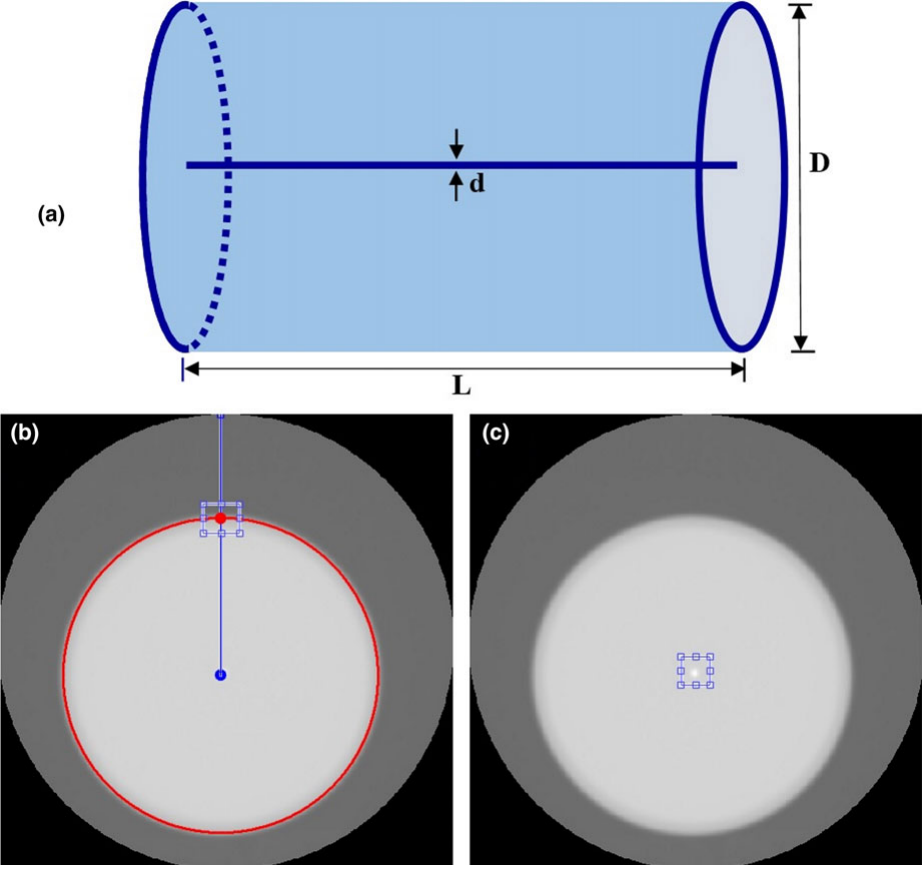
(a) The wire phantom comprised a cylinder of resin with a thin wire of diameter of 0.1 mm at the center. (b) ROI for MTF calculations using the proposed algorithm from the upper edge of the phantom. (c) ROI for MTF calculation with standard method obtained directly from the center of the image (point image).

To validate the MTF curves obtained using the edge phantom, we compared it with using a wire phantom as shown in Fig. 4(a). The wire phantom comprised a cylinder of resin (200 ml volume of a CT injector syringe, Kyorindo Nemoto Co., Ltd., Japan) with a diameter (*D*) of 4.8 cm. At the center of the phantom is a thin wire with a diameter (*d*) of 0.1 mm and a length (*L*) of about 5 cm. The phantom was filled with tap water to a volume of about 150 ml. The scan parameters of the wire phantom are indicated in Table 1. The FOV was 7.0 cm and the images were reconstructed using three filter types: FC13, FC30, and FC52.

The ROI for MTF calculations using the proposed algorithm from the upper edge of the phantom was shown in Fig. 4(b). A point image was obtained from the wire phantom and used to calculate MTF from the center of the phantom image with a rectangular ROI (32 × 32 pixels) as shown in Fig. 4(c). The image was cropped to that size and position. The pixels were then averaged along the *y*‐direction to obtain the profile of the pixel values at a point along the *x*‐direction or LSF curve, which was used to obtain the MTF curve with the same algorithm as in Section 2.A. The MTF results from the proposed method were then compared with the MTF values obtained directly from the point image.

### 2.D Validation of the automated MTF algorithm

To validate the automated MTF algorithm, we used one of the standard methods of MTF calculation, i.e. the ESF fitting method suggested by Boone and Seibert.^21^ This method was proposed for calculating MTF in digital mammography systems, but it can be used in CT systems with a standard filter, i.e. soft tissue kernel. In this method, the measured ESF is fitted to the equation:

(5) $$ESF^{+}\left(x\right)=a\left\{1-exp(b|x-x_{0}|)\right\}+c\;\text{erf}\left(d^{1/2}\left|x-x_{0}\right|\right)$$

The measured ESF curve is usually low to the left and high to the right, so that:

(6) $$ESF\left(x\right)=\begin{array}{cc} ESF^{+}\left(x\right) & \;\;\text{if}\;x\geq 0\\ -ESF^{+}\left(x\right) & \;\;\text{if}\;x\;<\;0 \end{array}$$

However, this does not take into account the offset and range of the measured ESF.^21^ Taking these into account the equation for fitting the ESF becomes:

(7) $$ESF_{\mathrm{fit}}\left(x\right)=e+f\cdot ESF\left(x\right)$$

**Figure 5:**
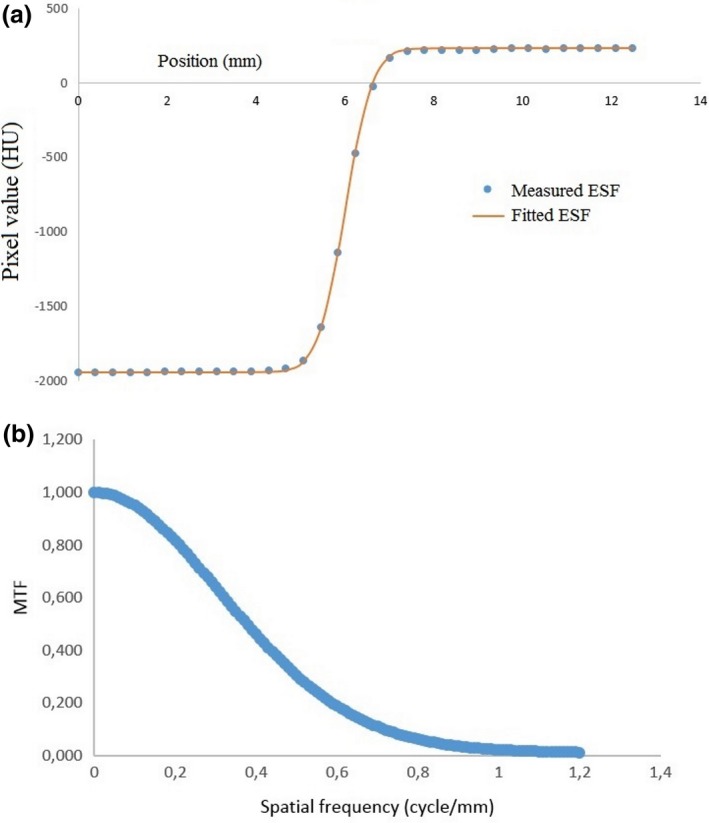
(a) The measured ESF is fitted by inputting the parameters **a**,** b**,** c**, and **d** by trial and error, to obtain the best correspondence to Eq. (7), and (b) The corresponding MTF curve calculated using Eq. (8).

A typical example fitting the measured ESF to Eq. (7) is shown in Fig. 5(a).

The fitting process between the new ESF curve and the measured ESF curve is carried out by choosing different values of **a**,** b**,** c**, and **d** by trial and error. After the ESF is accurately fitted, the coefficients of the fit (**a**,** b**,** c**, and **d**) were used to calculate the MTF using:

(8) $$MTF_{\mathrm{fit}}\left(f\right)=\frac{c\;exp\left(-\pi^{2}f^{2}/d\right)+\frac{a}{\left(1+4\pi^{2}f^{2}/b^{2}\right)}}{\left(c+a\right)}$$

An example of the resulting MTF is shown in Fig. 5(b).

## 3 RESULTS

### 3.A MTF validation using the edge phantom

**Figure 6:**
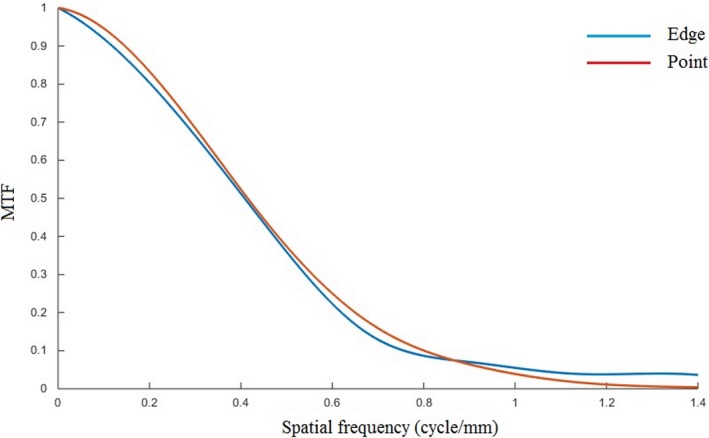
Comparison of MTF curves obtained using upper edge and central phantom (point image) for FC13 filter and FOV of 7.0 cm.

The MTF curves obtained using the upper edge and the wire phantom (point image) for FC13 and FOV of 7.0 cm are shown in Fig. 6. Both MTF curves are comparable. The spatial resolution values at 50% MTF are tabulated in Table 2. The differences in the 50% MTF values between the two approaches are within ±4% for all filters used (FC13, FC30 and FC52).

**Table 2:** The values of spatial resolution at 50% MTF from the upper edge and the center of the wire phantom (point image) for FC13, FC30 and FC52 filters, and for FOV of 7.0 cm

Filter	Spatial resolution at 50% MTF (cycle/mm)	
	Using edge image	Using point image
FC13	0.42 ± 0.01	0.42 ± 0.01
FC30	0.87 ± 0.04	0.85 ± 0.02
FC52	0.96 ± 0.06	0.92 ± 0.04

### 3.B Validation of automated MTF algorithm

**Figure 7:**
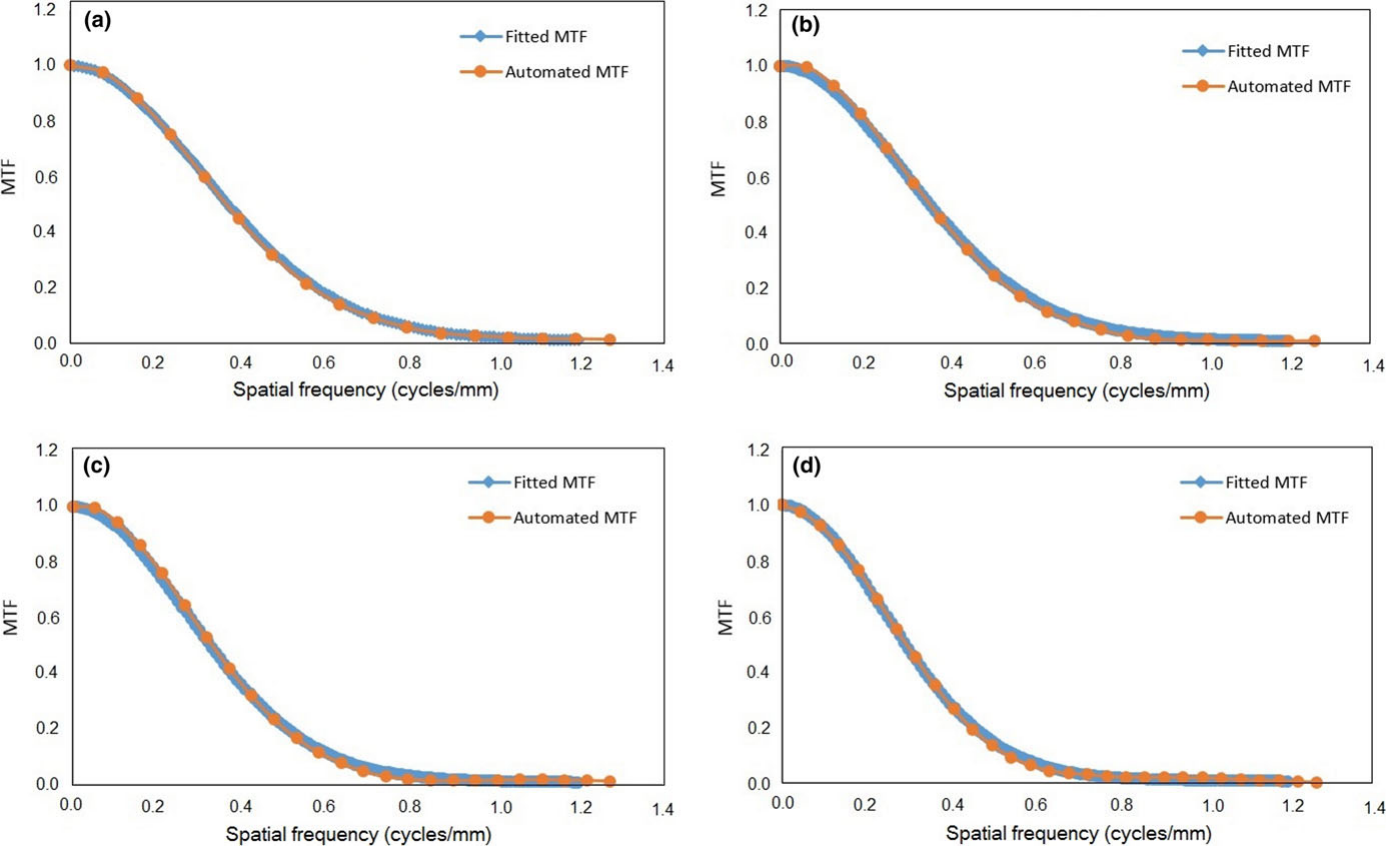
The results of the MTF curves from the automated and fitted methods for various FOVs. (a) 20 cm, (b) 25 cm, (c) 30 cm, and (d) 35 cm.

The best‐fit values of **a**,** b**,** c**,** d**, and *x*_0_, for various FOVs in the FC13 filter, are shown in Table 3. These values were then used to calculate the MTF curve using Eq. (8). The automated MTF curves were compared with those obtained by standard fitting of Fig. 7, for four different FOVs. The spatial resolution values at 50% MTF are tabulated in Table 4. The differences in the 50% MTF values between the automated method and the standard fitting method are within ±2%.

**Table 3:** The best‐fit values of **a**,** b**,** c**,** d**, and *x*_0_, for various FOVs in the FC13 filter

Parameter	FOV			
	20 cm	25 cm	30 cm	35 cm
**a**	1.30	1.30	1.30	1.30
**b**	2.25	2.05	1.85	1.65
**c**	8.70	8.70	8.70	8.70
**d**	2.00	1.85	1.55	1.25
*x*_0_	6.00	7.32	9.34	10.93

**Table 4:** The values of spatial resolution at 50% MTF from the automated calculation and the standard fitting methods for the head and body PMMA phantom, for various FOVs using the FC13 filter

FOV (cm)	Spatial resolution at 50% MTF (cycle/mm)	
	Automated calculation	Fitting method
20	0.37 ± 0.00	0.37
25	0.35 ± 0.00	0.36
30	0.33 ± 0.00	0.33
35	0.30 ± 0.00	0.29

### 3.C MTF for type filter variation

**Figure 8:**
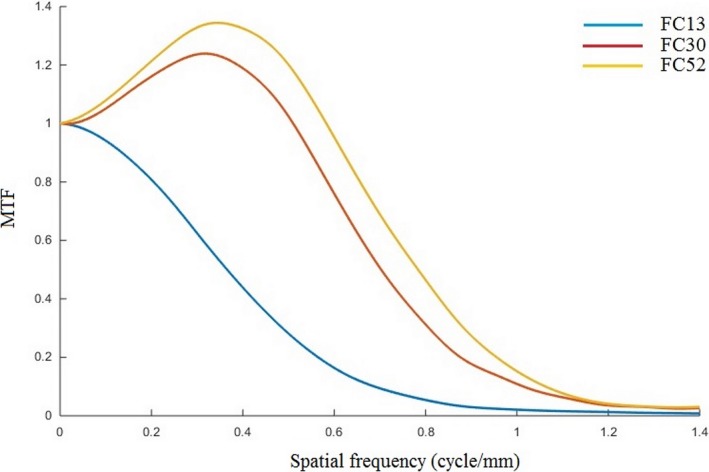
MTF curves for different filters (FC13, FC30 and FC52) using a FOV of 20 cm.

The MTF curves for the FC13, FC30, and FC52 filters at a FOV of 20 cm are shown in Fig. 8. They show that the automated MTF calculation is able to differentiate MTF curves for various filters. The largest spatial resolution is for the FC52 filter, followed by the FC30 filter, and the smallest spatial resolution is for the FC13 filter. The spatial resolution values (at 50% MTF) are 0.78 ± 0.01, 0.71 ± 0.01, and 0.37 ± 0.00 cycles/mm for the FC52, FC30, and FC13 filters, respectively.

### 3.D MTF for FOV variation

**Figure 9:**
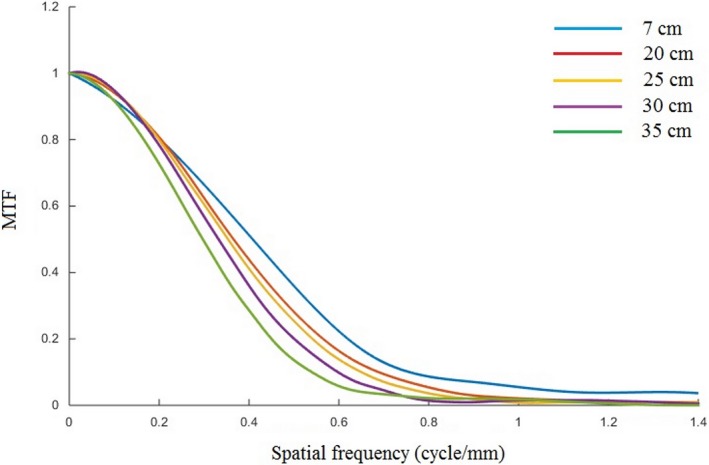
The MTF curves for different FOVs (7, 20, 25, 30, and 35 cm), using the FC13 filter.

**Figure 10:**
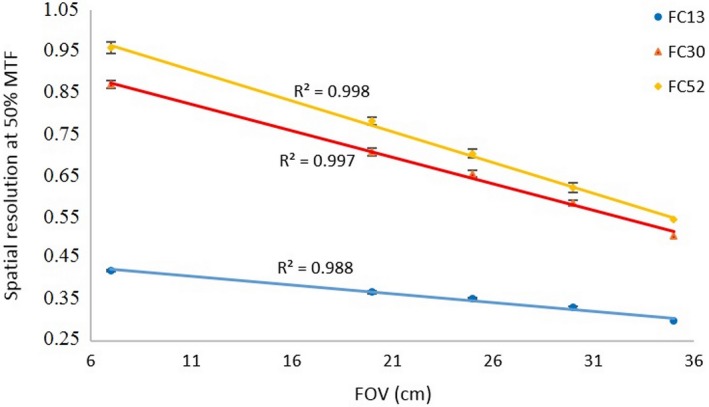
The spatial resolution (at 50% MTF) values for FOV variations for three type filters (FC13, FC30 and FC52).

The MTF curves for different FOVs using the FC13 filter are shown in Fig. 9. The spatial resolution gets smaller as the FOV increases (from 7 to 35 cm). The spatial resolution (at 50% MTF) values for different FOVs are shown in Fig. 10, for the three (FC13, FC30 and FC52) filters. The spatial resolution decreases linearly with increasing FOV (*R* ^2^ > 0.98 for all filters). Table 5 shows the spatial resolution values measured using both PMMA phantoms at FOV of 35 cm and for three types of reconstruction filters using our automated calculation. The spatial resolution values measured for both body and head PMMA phantoms are comparable to within ±4%; however, the standard deviations in the body phantom results are greater than in the head phantom due to noise.

**Table 5:** The values of spatial resolution at 50% MTF for the head and body PMMA phantom for various filters using a FOV of 35 cm

Filter type	Spatial resolution at 50% MTF (cycle/mm)	
	Head PMMA phantom	Body PMMA phantom
FC13	0.30 ± 0.00	0.31 ± 0.02
FC30	0.51 ± 0.01	0.50 ± 0.06
FC52	0.54 ± 0.01	0.55 ± 0.09

## 4 DISCUSSION

An algorithm for the automated calculation of MTF for PMMA phantom images has been developed and validated. The results agree well with values calculated using a standard fitting method developed by Boone and Seibert.^23^ The standard fitting method is straightforward and requires a simple computational calculation, but the fitting of the measured ESF curve is highly labor intensive. The complete automated MTF calculation take less than 1 s using a standard netbook. This proposed method may be applicable to all CT centers because it does not need a dedicated special phantom,^17^, ^18^, ^19^, ^20^, ^21^, ^22^, ^23^, ^24^, ^25^, ^26^ but rather the common PMMA phantom.

Unlike the standard fitting method^23^ which can only be applied to MTF calculations with certain types of filter reconstructions with soft‐tissue kernel, i.e. FC13, but is not applicable to other filters such as bone and lung kernels due to the additional contrast of each object edge, the automated MTF methods can be readily applied to all types of reconstruction filter. Figure 8 showed that the automated MTF calculation can distinguish the spatial resolution of images using soft tissue, bone, and lung kernels. It is likely that the proposed technique would help practitioners in medical centers to obtain the spatial resolution measurement for CT scans accurately, and independently of any subjectivity in measurement.

We confirmed that the spatial resolution of the head PMMA phantom decreases linearly (*R* ^2^ > 0.98) with increasing FOV. The spatial resolution of the body PMMA phantom was comparable to that measured in the head PMMA phantom for the same FOV (35 cm) (Table 5), although the standard deviation in the body phantom results are greater than in the head phantom. This is because, the noise in the body PMMA phantom is greater (about 6.5‐fold) than that in the head PMMA phantom at the same exposure factor (mAs and kVp), while the CT number in the head PMMA phantom (about 117 HU) is smaller than that in the body PMMA phantom (about 127 HU). Higher noise causes correspondingly higher MTF fluctuations, resulting in an increase in the standard deviation of the measured MTF.^23^, ^32^

The implication is that if in a clinical examination requires higher spatial resolution, the FOV value should be kept small. However, if the FOV is too small then the patient's image may be truncated.^33^ The selection of the FOV should be chosen carefully to match clinical needs.^34^, ^35^ Our study had several limitations. We only used one scanner model. We implemented the method using code written in MatLab, which would need to be installed at each medical center. The complete code for our automated MTF calculation can be found in the Data S1.

## 5 CONCLUSIONS

A method for calculating the spatial resolution automatically using an edge phantom (viz., the PMMA phantom) has been developed. The method is very easy to implement in clinical applications, works very quickly, and is more objective since it is not influenced by user experience. We validated the automated MTF calculation using an edge of phantom by comparing the results with standard methods. The 50% MTF values obtained from the edge and point phantoms agreed to within ±4%, and the difference between the automated and fitted method was within ±2%. We found that spatial resolution decreases linearly with increasing FOV (*R* ^2^ > 0.98).

## CONFLICT OF INTEREST

The authors declare no conflicts of interest.

## Supporting information


**Data S1**. Codes of the automated MTF calculation.
