# Supplementary material for: An algorithm for automated modulation transfer function measurement using an edge of a PMMA phantom: Impact of field of view on spatial resolution of CT images
Source: J Appl Clin Med Phys. 2018 Oct 19;19(6):244–52. doi: 10.1002/acm2.12476 (PMC6236841; doi:10.1002/acm2.12476)
Supplement: Supplementary file 1 — Data S1. Codes of the automated MTF calculation. [file ACM2-19-244-s001.pdf]

```
%------------------------------------------------------------
% Automated MTF calculation using CTDI phantom
% Developed by Choirul Anam (Diponegoro University)
%
% DIRECTION: This code should be copied in the Matlab Editor.
% Then, file name of image in the "Open CT image" part should
% be edited in accordance with the file name of CT image.
% In this example, the file name is "CTDIphantom.dcm".
% The CT image should be saved in the same folder of this code.
% ------------------------------------------------------------

% ------------------------------------------------------------
% Celar computer memories and screen
clear all
clc
% ------------------------------------------------------------

% ------------------------------------------------------------
% Open CT image
Citra = dicomread('CTDIphantom.dcm');%Please edit this file name
info = dicominfo('CTDIphantom.dcm'); %Please edit this file name
Potong=info.RescaleIntercept;
Miring=info.RescaleSlope;
FV=info.ReconstructionDiameter;
Spasi=info.PixelSpacing;
imshow(Citra,'DisplayRange',[]);
% ------------------------------------------------------------

% ------------------------------------------------------------
% Transform CT data to HU
[a b]=size(Citra);
D1=int16(Citra);
D2=zeros(a,b);
D2=int16(D2);
for k=1:a
    for l=1:b
        D2(k,l)=D1(k,l)*Miring+Potong;
    end
end
figure(1)
imshow(D2,'DisplayRange',[]);
% ------------------------------------------------------------

% ------------------------------------------------------------
% Automatic segmentation
B=zeros(a,b);
for i=1:a
    for j=1:b
        if D2(i,j) < (800-1024)
            B(i,j)=0;
        else
            B(i,j)=1;
        end
     end
end
Obyek=sum(sum(B));
```

```
if Obyek > 1
   BR = bwmorph(B,'remove');
   BL=bwlabel(BR);
   NM=max(max(BL));
   for put=1:NM
       CB=zeros(a,b);
       for i=1:a
           for j=1:b
               if BL(i,j) == put
                   CB(i,j)=1;
               end
           end
       end
   DB = imfill(CB,'holes');
   LA(put)=bwarea(DB);
   end
   terbesar=max(LA);
   for iter=1:NM
       if (LA(iter)==terbesar)
           urutan =iter;
       end
   end
   CB=zeros(a,b);
   for i=1:a
       for j=1:b
           if BL(i,j) == urutan
               CB(i,j)=1;
           end
       end
   end
   DB = imfill(CB,'holes');
else
   DB=zeros(a,b);
end
figure(2)
imshow(D2,'DisplayRange',[]);
DBP = bwmorph(DB,'remove');
[n m]=size(DBP);
p=0;
for i=1:n
    for j=1:m
     if DBP(i,j)==1
         p=p+1;
         x(p)=j;
         y(p)=i;
     end
    end
end
hold on
scatter(x,y,'r', '.')
% -------------------------------------------------------------

% -------------------------------------------------------------
% Determination center of the phantom image
x0=round(a/2); y0=round(b/2);
N=0;
xpos=0; ypos=0;
```

```
for i=1:a
    for j=1:b
        if DB(i,j) > 0
            N=N+1;
            xpos=xpos+i;
            ypos=ypos+j;
        end
    end
end
yb=round(xpos/N);
xb=round(ypos/N);
plot(xb,yb,'b.', 'MarkerSize', 30)
% ------------------------------------------------------------

% ------------------------------------------------------------
% ROI determination
n=16; %Height of ROI
m=20;  %Width of ROI
[p q]=size(x);
j=0;
for i=1:q
    if x(i)==xb
        j=j+1;
        yc(j)=y(i);
        ke(j)=i;
    end
end
yd=min(yc);
num=min(ke);
h =imline(gca, [xb xb], [1 yb]); setColor(h, 'b');
h = imrect(gca, [xb-m yd-n 2*m+1 2*n+1]);
plot(xb,yd,'r.', 'MarkerSize', 30)
% ------------------------------------------------------------

% ------------------------------------------------------------
% ESF determination and tail replacement
figure(3)
hold on
krop1=Citra(yd-n:yd+n, xb-m:xb+m);
krop2=krop1';
krop3=(sum(krop2))/(m+1);
krop=krop3';
sel=zeros(2*n+1,1);
k=0;
for i=2:2*n+1
    take=krop(i);
    sel(i)=abs((krop(i)-krop(i-1))*100/(krop(i)));
    if sel(i)>=10
        k=k+1;
        mulainaik(k)=i-3;
    end
end
mulai=min(mulainaik);
depan=fliplr(krop(1:mulai));
j=0;
for i=(2*n+1-mulai+1):2*n+1
```

```
    j=j+1;
    krop(i)=krop(2*n+1-mulai+1)*(depan(j)/depan(1));
end
% ------------------------------------------------------------

% ------------------------------------------------------------
% ESF Interpolation
interpolasi=4;
dinter=1/interpolasi;
nn=0:1:2*n;
nnn=0:dinter:2*n;
krop2=spline(nn(1:2*n+1),krop(1:2*n+1),nnn(1:interpolasi*2*n+1));
krop=krop2;
n=interpolasi*n;
spaki=Spasi(1,1)*dinter;
sumbuX=0:spaki:2*n*spaki;
plot(sumbuX, krop, 'LineWidth',2);
xlabel('Position (mm)')
ylabel('Pixel value (HU)')
set(gcf, 'color', 'w')
hold off
% ------------------------------------------------------------

% ------------------------------------------------------------
% LSF Calculation, zeroing and normalization process
c=krop;
Turunan=diff(c);
Xc=Turunan;
[a b]=size(Xc)
Xca=sum(Xc(1:5))/5;
for i=1:b
    Xc(i)=Xc(i)-Xca;
end
Xd=sum(Xc); Xd=Xc*(1/Xd);
figure(4)
sumbuXX=spaki:spaki:b*spaki;
plot(sumbuXX, Xd, 'LineWidth',2)
xlabel('Position (mm)')
ylabel('LSF')
set(gcf, 'color', 'w')
% ------------------------------------------------------------

% ------------------------------------------------------------
% MTF Calculation
Xe=fftshift(abs(fft(Xd)));
figure(5)
[c d]=size(Xe)
tengah=round(d/2);
Xee=Xe(1,tengah+1:d);
[f g]=size(Xee);
SpatialFrequency=(1/((2*n+1)*spaki));
sumbuXXX=0:SpatialFrequency:(g-1)*SpatialFrequency;
plot(sumbuXXX,Xee, 'LineWidth',2)
xlabel('Spatial frequency (cycles/mm)')
ylabel('MTF')
set(gcf, 'color', 'w')
```

```
xlim([0 1.4]);
MTF50=interp1(Xee, sumbuXXX, 0.5, 'linear')
% ------------------------------------------------------------
```
